# Effect of Stem Cell-Derived Extracellular Vesicles on Damaged Human Corneal Endothelial Cells

**DOI:** 10.1155/2021/6644463

**Published:** 2021-01-16

**Authors:** Raffaele Nuzzi, Lola Buono, Simona Scalabrin, Marco De Iuliis, Benedetta Bussolati

**Affiliations:** ^1^Eye Clinic, Department of Surgical Sciences, University of Turin, AOU Città della Salute e della Scienza, Turin, Italy; ^2^Department of Biotechnology and Health Sciences, University of Turin, Turin, Italy

## Abstract

**Purpose:**

Human corneal endothelial cells (HCECs) are essential to visual function; however, since they have limited proliferative capacity *in vivo*, they are prone to corneal endothelial dysfunction. At present, the only treatment is a corneal transplantation from donor cadavers. Also, due to a global shortage of donor corneas, it is important to find alternative strategies. Recent studies highlight that stem cell–derived extracellular vesicles (EVs) play a relevant role in stem cell-induced regeneration by reprogramming injured cells and inducing proregenerative pathways. The aim of this work is to evaluate whether EVs derived from mesenchymal stem cells (MSC-EVs) are able to promote regeneration of damaged HCECs.

**Methods:**

We isolated HCECs from discarded corneas in patients undergoing corneal transplantation or enucleation (*N* = 23 patients). Bone marrow mesenchymal stem cells (MSCs) were obtained from Lonza, cultured, and characterized. MSC-EVs were obtained from supernatants of MSCs. In order to establish a valid *in vitro* damage model to test the regenerative potential of EVs on HCECs, we evaluated the proliferation rate and the apoptosis after exposing the cells to serum-deprived medium at different concentrations for 24 hours. We then evaluated the HCEC migration through a wound healing assay.

**Results:**

In the selected serum deprivation damage conditions, the treatment with different doses of MSC-EVs resulted in a significantly higher proliferation rate of HCECs at all the tested concentrations of EVs (5‐20 × 10^3^ MSC-EV/cell). MSC-EVs/cell induced a significant decrease in number of total apoptotic cells after 24 hours of serum deprivation. Finally, the wound healing assay showed a significantly faster repair of the wound after HCEC treatment with MSC-EVs.

**Conclusions:**

Results highlight the already well-known proregenerative potential of MSC-EVs in a totally new biological model, the endothelium of the cornea. MSC-EVs, indeed, induced proliferation and survival of HCECs, promoting the migration of HCECs *in vitro*.

## 1. Introduction

Corneal transparency is the result of many factors such as its structural anatomy and the physiology of its components [1]. The cornea is a fine example of natural engineering: as any accumulation of fluid would affect stromal transparency and health, a mechanism for maintaining stromal deturgescence is required. This role is up to endothelium. Human corneal endothelial cells (HCECs) can do their job by functioning both as a barrier to fluid movement into the cornea and an active pump that moves ions and draws water osmotically, from the stroma into the aqueous humor [2, 3]. The combined leaky barrier and fluid pump is called a pump-leak mechanism [4].

Since endothelial cells have no mitotic activity in vivo (although they can be induced to divide in cultured corneas) [5, 6], HCECs are prone to corneal endothelial dysfunction that eventually could lead to blindness. However, the human corneas at birth are characterized by a considerable endothelial cells reserve; in fact during the first months of life, endothelial cell density is around 6.000 cells/mm^2^, while during eighth decades of life, endothelial cell density is approximately 2.600 cells/mm^2^ [7, 8], while the percentage of cells with a hexagonal shape decreases from 75% to 60% [9]. The causes of endothelial cell decrease are age but also trauma, inflammation, corneal disease, and surgical procedures.

There are few diseases affecting primary the corneal endothelium [10]. Fuchs' endothelial corneal dystrophy (FECD) is the most common. In FECD, the pump function of the endothelial cells decreases, followed by a reduced barrier function [11] so that the endothelium becomes unable to maintain fluid balance and consequently corneal clarity. Other endothelial diseases are posterior polymorphous dystrophy [12], congenital hereditary endothelial dystrophy [13, 14], and iridocorneal endothelial syndrome [15]. Moreover, being the cornea the outer part of the eye, it is most likely to sustain damage due to various sources of stress, causing secondary corneal endotheliopathies. Main kinds of insults are external trauma; metabolic damage, such as hypoxia, for example, caused by contact lens wear [16], or hyperglycemia; toxic, for example, due to drugs or their preservatives; related to alterations in pH or osmolarity; and associated to surgical procedures, in particular related to phacoemulsification and corneal transplantation [17–19].

The wound healing of endothelium has certain proper characteristics: endothelium mostly heals by cell migration and increased cell spreading and may undergo epithelial-mesenchymal transformation in this process, but cell proliferation plays a secondary role [20]. More in detail, the process of resurfacing an injured area is characterized by 3 steps. Endothelial cells do not undergo mitosis in vivo, so in the first step, the injured cells have to be quickly replaced by migration and extension of the adjacent surviving cells, in order to form a temporary barrier with reduced pump activity. So, surviving cells have the ability to “stretch” over the space of the degenerated cells, growing in size (polymegathism) and losing their typical hexagonal shape (pleomorphism). Endothelial wound healing is associated with a transient acquisition of fibroblastic morphology and actin stress fibers by migrating cells, which is consistent with endothelial-mesenchymal transformation [21] [22]. In the second stage, the number of tight junctions and pump sites returns to normal levels; the endothelial cells form irregular polygons; the corneal thickness typically returns to normal, and transparency is restored. The third stage that can last for many months involves remodeling of the endothelial cells to form more regular hexagonal shapes. Anyway, the endothelium has a limited response to stress: mild injury may result only in changes in cell size and shape, while greater stress may result in cell loss that leads to irreversible alterations in the endothelial morphology and biological function [23]. When endothelial cell decreases below 500 cells/mm^2^, the eye becomes at risk for the development of corneal edema, with loss of corneal clarity [24].

Whatever it is the mechanism of damage, at present, the only treatment for this dysfunction is a corneal transplantation, or keratoplasty, from donor cadavers. In keratoplasty, a wide part of the central cornea is replaced with a graft, an allogeneic cornea which has been removed post mortem from a donor. It can be classified on the basis of the transplantation technique [25]: from the replacement of full-thickness cornea with a healthy donor cornea, called full-thickness penetrating keratoplasty (PKP), to the replacement of only selective diseased layers, called partial lamellar corneal keratoplasty. The last one can be divided in anterior lamellar keratoplasty or posterior lamellar keratoplasty (PLK). In case of endothelial pathology, PKP or PLK may be indicated. Despite recent improvements in surgery techniques, corneal transplantation presents some limits and difficulties. The surgical techniques are hard to perform, and complications are possible, including choroidal haemorrhage, glaucoma, loose sutures, suture infiltrates, suture-associated astigmatism, dislocation of the graft, and graft infection [26–29]. The visual outcome is generally good, but the graft failure is not uncommon, making necessary a long-term immunosuppressive topical therapy [30, 31]. Anyway, the main problems of keratoplasty are represented by the necessity of a graft from a donor and by the legal, ethical and cultural issues related to the complex process of organ donation and transplantation. It is fundamental a good coordination between medical officers, nurses, technicians, forensic experts, and the legal system in order to support graft availability. The whole process is not easy, and the need for donor corneas is increasing, contributing to a demand-supply shortage, especially in developing countries.

Due to keratoplasty limits and complications, along with a global shortage of donor corneas, it is important to find alternative strategies of treatment in order to overcome corneal transplantation. Considering all these issues, different strategies have been studied.

Recent studies highlight that stem cell–derived extracellular vesicles (EVs) play a relevant role in stem cell-induced regeneration by reprogramming injured cells and by inducing proregenerative pathways. A particularly promising area of investigation seems to be the use of extracellular vesicles derived by mesenchymal stem cells (MSC-EVs) that could be able to promote regeneration of damaged endothelium. MSC-EVs have been widely studied in various disease models [32, 33], and in the last decade, they have been of interest in many ophthalmologic pathologies[34, 35]. In 2018, indeed, it was evaluated the effect of MSC-EVs on corneal wound healing, and it was shown that human corneal MSC-EVs significantly increased the proliferation of human corneal epithelial cells in vitro and accelerated corneal wound closure in a murine epithelial mechanical injury model [36]. The aim of our study is, therefore, to investigate whether EVs, released by human bone marrow MSCs, may be beneficial in reducing ER-stress and HCEC apoptosis induced by an in vitro damage model caused by nutrient deprivation.

## 2. Material and Methods

### 2.1. Isolation and Characterization of HCECs

We isolated HCECs from discarded cornea patients undergoing corneal transplantation or enucleation (*N* = 23 patients) due to different pathologies (Table 1).

Briefly, the Descemet's membrane and corneal endothelial cells were stripped from the posterior surface of the peripheral corneoscleral tissue using a scalp and afterwards digested with collagenase A (2 mg/ml). The digested membrane and cells (HCECs) were then placed on a Petri dish previously coated with fibronectin; HCECs migrated out of the Descemet's membrane and were maintained at 37°C in a humidified atmosphere of 95% air and 5% CO2 and cultured in ENDOGRO 10% FBS for seven passages. Once cells reached confluency, they were passaged at 1 : 2 ratios using 0.25% trypsin 0.02% ethylenediaminetetraacetic acid solution (Sigma-Aldrich, USA).

**Figure 1:**
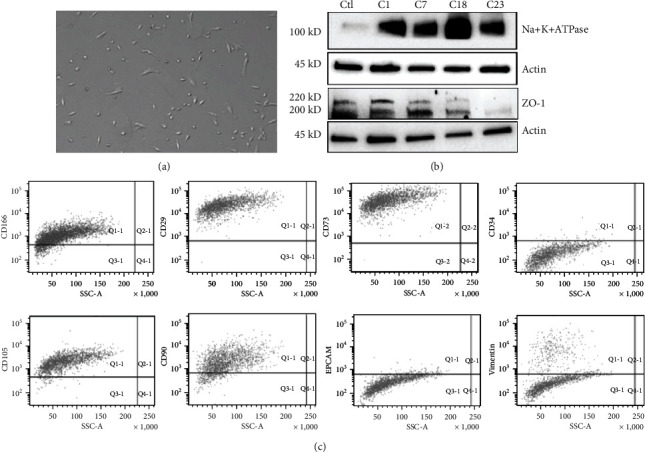
Isolation and characterization of corneal endothelial cells. (a) Representative micrograph of HCEC at passage 3. (b) Representative micrographs of western blot on four HCEC independent cell lines deriving from different patients (C1, C7, C18, and C23). The control (Ctl) is represented by the renal HK2 cell line, negative for Na + K + ATPase and positive for ZO-1. Actin was used as an endogenous loading reference. (c) Representative flow cytometry analysis of HCECs showing the expression of CD166, CD105, CD29, CD90, CD73, CD34, EPCAM, and Vimentin on HCECs.

From each patient we isolated a cell line of HCECs, each cell line was kept in culture up to the sixth passage; all experiments were performed between passages 2 and 4 (Figure 1(a)). HCECs deriving from three independent cell lines were then characterized by the expression of HCEC main marker ATP1A1 (Na + K + ATPase) and ZO-1 (Figure 1(b)) and by presence of the surface markers CD166 [37], CD105, CD29 [38], CD90, and CD73 and by the absence of CD34 [39], of the epithelial marker EPCAM, and of the stromal marker Vimentin (Figure 1(c)).

For protein analysis, HCECs were lysed at 4°C for 30 min in RIPA buffer (20 nM Tris·HCl, 150 nM NaCl, 1% deoxycholate, 0.1% SDS, 1% Triton X-100, pH 7.8) supplemented with protease and phosphatase inhibitor cocktail and PMSF (Sigma-Aldrich). Aliquots of the cell lysates containing 25 *μ*g protein, as determined by the Bradford method, were run on 4-20% (BioRad) SDS-PAGE under reducing conditions and blotted onto PVDF membrane filters using the iBLOT system (Life Technologies). For western blot analysis, Na + K + ATPase (Abcam), ZO-1 (Invitrogen), and Actin (Santa-Cruz Biotechnology) antibodies were used for the characterization of HCECs.

For cytofluorimetric analysis, cells were detached using a nonenzymatic cell dissociation solution (Sigma-Aldrich) and resuspended in PBS 0.1% BSA (Sigma-Aldrich) and incubated with antibodies. The following antibodies, conjugated with fluorescein isothiocyanate (FITC), phycoerythrin (PE), or allophycocyanin (APC), were used: CD166, CD34 (BD Biosciences), EPCAM, Vimentin, CD90, CD105, CD73, and CD29 (Miltenyi Biotech).

### 2.2. Isolation and Characterization of MSC-EVs

**Figure 2:**
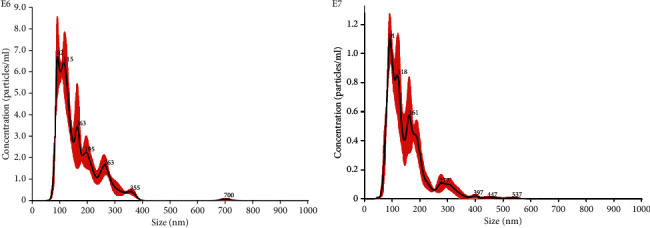
EV quantification using Nanosight Nanoparticle Tracking Analysis. MSC produced EVs in cell culture with a mean size of 163.3 nm, with a homogenous EV population.

MSC-EVs were obtained as previously described [40]. In brief, bone marrow mesenchymal stem cells (MSCs) were obtained from Lonza, cultured, and characterized [41]. MSCs derived from five preparations were used up to the sixth passage of culture. MSC-EVs were obtained from supernatants of MSCs cultured overnight in RPMI deprived of FCS. After removal of cell debris and apoptotic bodies by centrifugation at 3000 g for 20 min, EVs were purified by 2 h ultracentrifugation at 100,000 g at 4°C (Beckman Coulter Optima L-90 K; Fullerton). EVs were used freshly or stored at -80°C after resuspension in RPMI supplemented with 1% dimethyl sulfoxide (DMSO). Analysis of size distribution and enumeration of EVs were performed using NanoSight LM10 (NanoSight Ltd.) equipped with a 405 nm laser and the Nanoparticle Tracking Analysis (NTA) 2.3 software (Figure 2).

### 2.3. Establishing a Serum Deprivation Damage Model on HCECs

**Figure 3:**
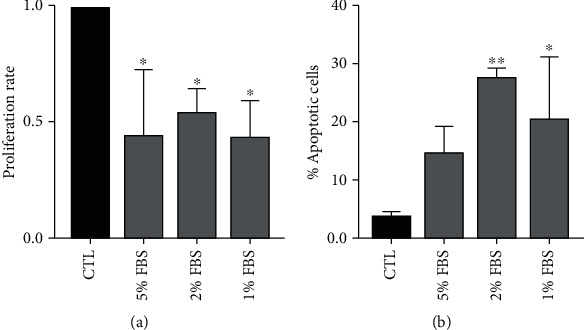
Damage setting by serum deprivation of HCECs. (a) Proliferation levels at different concentration of FBS (5-1%) of HCECs after 24 h of treatment. Data are represented as mean ± SD of three independent experiments normalized to CTL. (b) Percentage of apoptotic HCECs at different concentration of FBS (5-1%) of HCECs after 24 h of treatment. Data are represented as mean ± SD of three independent experiments normalized to CTL. One-way ANOVA analysis with Tukey's multicomparison tests was performed among FBS and CTL (^∗^*p* < 0.05, ^∗∗^*p* < 0.001).

In this study, serum deprivation culture was used to mimic the nutrient deficient environment in order to establish a valid *in vitro* damage model to test the regenerative potential of EVs on HCECs. We evaluated the proliferation rate and the apoptosis after exposing the cells to serum-deprived medium at different concentrations for 24 hours (Figure 3). Serum deprivation significantly inhibited HCEC proliferation in all the different concentrations of FBS (Figure 3(a)), and the survival of HCECs was inhibited at both 1% and 2% FBS presence (Figure 3(b)). We chose the 2% FBS concentration to go on with the experiments.

### 2.4. Evaluation of Regenerative Potential of MSC-EVs: Proliferation and Apoptosis Assay

For proliferation assay, cells were plated in growth medium at a concentration of 5000 HCEC-cells/well in a 96-multiwell plate and left adhere overnight. The day after the culture medium was removed and a new medium containing different concentrations of FBS (1-5%) was added to the cells to induce a damage. After 24 hours of serum deprivation, differential concentrations of MSC-EVs (5‐20 × 10^3^ MSC-EV/cell) were added to the medium for further 24 hours. DNA synthesis was detected after 4 hours of incorporation of 5-bromo-2-deoxyuridine (BrdU) using an enzyme-linked assay kit (Chemicon). Data are expressed as the mean ± SD of the media of absorbance of at least three different experiments, normalized to control (not treated cells).

To evaluate apoptosis, Annexin V assay was performed using the MuseTM Annexin V and Dead Cell Kit (Millipore), according to the manufacturer's recommendations and following the methods of Brossa et al. [42]. Briefly, 30 × 10^3^ cells in a 24-well plate were incubated with different concentrations of FBS for 24 hours, and different amount of MSC-EVs was added to the medium for further 24 hours. Cells were then detached and resuspended in MuseTM Annexin V and Dead Cell Kit, and the percentage of apoptotic cells (Annexin V+) was detected.

### 2.5. Evaluation of HCEC Migration: Wound Healing Assay

For the wound-healing migration assay, 30 × 10^3^ HCECs in a 24-well plate kept in damage conditions for 24 hours and treated for further 24 hours with 10 × 10^3^ MSC-EV/cell (2% FBS) were scratched using a 10 *μ*l pipette tip once the cell confluence reached approximately 90%. Then, the detached cells were washed and removed. Representative photographs were taken under a light microscope (Olympus Life Science) at 0 h and 6 h after wounding. The scratch length was measured three times for each photograph; 10 photographs per condition were taken.

### 2.6. Statistical Analysis

Statistical analysis was performed by using one-way ANOVA with Tukey's multicomparison tests, with a single pooled variance. A *p* value of *p* < 0.05 was considered significant.

## 3. Results

### 3.1. MSC-EVs Induce Proliferation and Survival of HCECs

**Figure 4:**
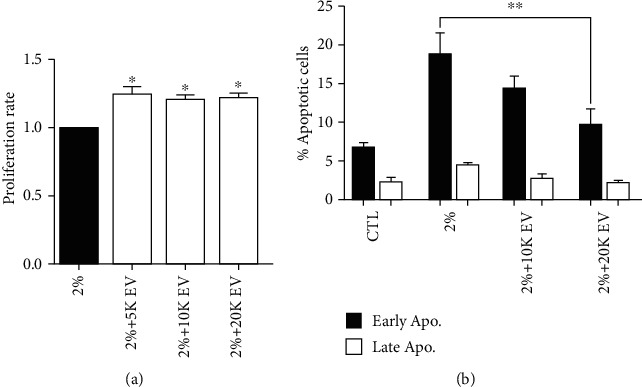
Proliferation and apoptosis of HCECs in damage conditions, treated with different doses of MSC-EVs. (a). Proliferation levels of HCECs maintained in 2% FBS for 24 hours and treated for further 24 hours with different doses of MSC-EVs (5‐20 × 10^3^ MSC-EV/cell). Data are represented as mean ± SD of four independent experiments normalized to CTL. (b). Percentage of early apoptotic (black columns) and late apoptotic (grey column) HCECs cultured either in ENDOGRO 10% (CTL) or in 2% FBS for 24 hours (2%) and treated for further 24 hours with different doses of MSC-EVs (10 and 20 × 10^3^ MSC-EV/cell). One-way ANOVA analysis with Tukey's multicomparison test was performed among 2% FBS alone and 2% + EVs (^∗^*p* < 0.05, ^∗∗^*p* < 0.001).

In the selected serum deprivation damage conditions, the treatment with different doses of MSC-EVs resulted in a significantly higher proliferation rate of HCECs at all the tested concentrations of EVs (5‐20 × 10^3^ MSC-EV/cell) (Figure 4(a)). We then evaluated the percentage of apoptotic cells after serum deprivation and following the treatment with MSC-EVs. 20 × 10^3^ MSC-EVs/cell induced a significant decrease in number of total apoptotic cells (Figure 4(b)) after 24 hours of serum deprivation (Figure 4).

### 3.2. MSC-EVs Induce HCECs Migration

**Figure 5:**
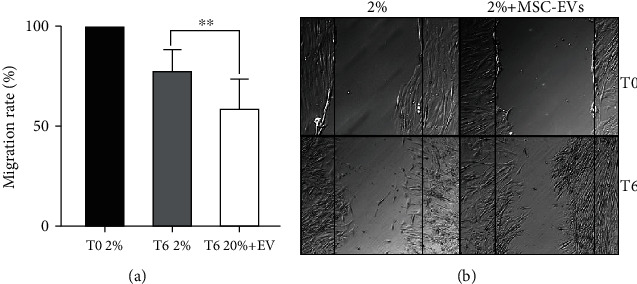
Wound healing assay of HCECs in damage conditions treated with MSC-EVs. (a) Wound healing assays were performed to evaluate cell motility of the HCECs in damage conditions (2% FBS); the migration rate was measured by the length of the scratch at different timings (T0 and T6), in absence (T6 2%) or in presence of 20 × 10^3^ MSC-EV/cell (T6 2% + EV). Data are represented as mean ± SD of nine independent experiments normalized to T0 2%. One-way ANOVA analysis with Tukey's multicomparison test was performed among 2% FBS and 2% + EVs (^∗∗^*p* < 0.001). (b) Representative pictures of HCECs in damage conditions (2% FBS) cultured in the presence or absence of 20 × 10^3^ MSC-EV/cell (2% + EV). Pictures were taken at time 0 (T0) and after 6 hours from the scratch (T6).

The wound healing assay showed a significantly faster repair of the wound after HCEC treatment with MSC-EVs (10 × 10^3^ EV/cell) in the serum deprivation model. We can see in Figure 5 that in damage conditions, after 6 hours from the scratch in presence of MSC-EVs (T6 2% + MSC − EVs), the length of the scratch is significantly shorter than the scratch in absence of MSC-EVs (T6 2%).

## 4. Discussion

MSCs, isolated in many tissues, have been originally described as multipotent stem cells with the potential to differentiate into different cell types [43]. This ability represented the initial drive for their therapeutics use, but this original rationale has then become gradually weaker. In fact, many studies reported that despite functional improvement after MSC transplantation, MSC engraftment and differentiation into proper cell types were infrequent [44, 45]. Moreover, efficacy of MSCs did not seem to be dependent on the physical proximity of the transplanted cells to the target tissue [46]. These evidences led to the idea that MSCs may exert their therapeutic effects thanks to a paracrine action [47]. Nowadays, MSCs are increasingly seen as stromal support progenitor cells with the potential to differentiate into stromal support cells and secrete factors able to limit cellular injury to support the stroma or other cells, by maintaining a microenvironmental niche that equilibrate the quiescence of stem [48, 49]. At the beginning, efforts to characterize MSC secretion focused on small molecules such as growth factors, chemokines, and cytokines, but no one could sufficiently account for the efficacy of MSCs, until a study conducted in 2009 demonstrated that microvesicles, a class of EVs, secreted by MSCs protected against acute tubular injury [50]. Since then, EVs have been more and more reported as the therapeutic driving force of MSCs. MSCs have been reported to secrete different classes of EVs: microvesicles, microparticles, and exosomes [51, 52]. These classes are currently defined by physical and biological parameters [53]. However many criteria are not exclusive to any specific class so that the presence of distinct biological entities is not sure [54].

EVs act as one-way conveyors of cellular material, including nucleic acids, proteins, and lipids, from a secreting cell to a target cell to modulate its activity. They represent an intercellular communication vehicle secreted by MSCs to exert a stromal support function by regulating cellular processes such as communication, structure and mechanics, inflammation, tissue repair and regeneration, and metabolism. In addition, EVs are able to mediate MSC interactions with several cell types in immediate but also remote areas; in fact thanks to their biophysical features, they can be easily transported through the blood and other biological fluids, acting in para- and endocrine manner. Their final aim is the maintenance of a dynamic and homeostatic microenvironment. This role is particularly important when tissue microenvironment is altered by an injury or a disease [55]. As a consequence, extensive research is focusing on the potential therapeutic applications of EVs in several areas [56]. More in general, the idea of MSC-EVs as stromal support mediators provides a rationale for the therapeutic efficacy of MSCs and their secretions in a wide spectrum of diseases, also in ophthalmic area, in order to restore tissue homeostasis and enhance tissues recovery, reparation, and regeneration.

Many functions of EVs have been identified. Currently, the best described stromal support function of MSC secretions is the preservation of hematopoietic stem cell homeostasis [57] and the tumor microenvironment [58]. On the other hand, investigations about EV functions in many specialized tissues of the eye are just at the beginning. However, the etiology of several eye pathologies, including age-related macular degeneration (AMD), diabetic retinopathy, glaucoma, and corneal angiogenesis, involves activation of immune cells, inflammation, fibrosis, cell degeneration, and neovascularisation, with exosomes being probable mediators of these processes. As a consequence, their employment may be a therapeutic strategy for such eye disorders [59–66]. Cell death is part of the pathology process in all eye diseases, for example, trabecular meshwork (TM) cells and retinal ganglion cells (RGCs) in glaucoma [67, 68], retinal pigment epithelium cells and photoreceptors in AMD with geographic atrophy [69], and corneal surface and endothelial cells in corneal diseases [70], so stem cell-based strategies are being studied in order to obtain cell replacement [71–73]. In practice, up to date, stem cell therapies have shown limited success. For example, differentiation of stem cells into RGC-like cells has only been accomplished in culture [74]. In addition, another limit of this strategy is the potential tumorigenic and immunogenic risks [75].

Anyway, as discussed before, being the main therapeutic effect of stem cells probably due to their paracrine action [76], another strategy may be exosome employment that may have several possible applications in ophthalmic pathology. Scientific research has recently focused on the use of exosomes derived from MSCs in various models of retinal damage in vitro and in vivo as they, compared to MSCs, have similar functions and at the same time have different advantages such as greater stability and handling, a lower chance of immunological rejection, and no risk of malignant transformation, being a cell-free approach. The treatment of pathologies and retinal damage with MSC-EVs typically takes place through intravitreal injection, allowing their direct action on the retinal cells and avoiding potential adverse effects towards other organs. Their use has been successfully studied in several diseases of the retina, such as retinal cell degeneration, refractory macular holes, and retinal detachments [35–77]. In all these cases, EVs have demonstrated a therapeutic effect, encouraging the realization of further studies. Exosomes from MSCs may also be able to enhance neural repair, and this action may be useful to restore retinal ganglion cell glaucoma [78]. They can also stimulate proliferation in a number cell types [79, 80], being a possible strategy to induce proliferation of TM cells in glaucoma [81]. Finally, the immunomodulatory effects of exosomes could be used to decrease inflammation and fibrosis for treatment of inflammatory eye diseases [82, 83] but also dry eye disease mediated by activation of immune cells [84].

Nevertheless, research about EVs in corneal pathology has been limited. Among corneal pathologies, Samaeekia et al. recently evaluated the effect of MSC-derived EVs on corneal wound healing and showed that human corneal MSC-EVs significantly increased the proliferation of human corneal epithelial cells *in vitro* and accelerated corneal wound closure *in vivo*^36^. Moreover, it was recently reported that the coculture of corneal stromal cells with MSC-EVs resulted in enhanced viability and proliferative ability and increased plasticity [85]. Starting from this preexisting data, we established an *in vitro* model to study the effect of MSC-EVs on a different corneal layer, the corneal endothelium.

In our work, the results highlight the already well-known proregenerative potential of MSC-EVs in a totally new biological model. In the selected serum deprivation damage conditions, the treatment with different doses of MSC-EVs resulted in a significantly higher proliferation rate of HCECs at all the tested concentrations of EVs. MSC-EVs/cell induced a significant decrease in number of total apoptotic cells after 24 hours of serum deprivation. Finally, the wound healing assay showed a significantly faster repair of the wound after HCEC treatment with MSC-EVs.

Our group is working on further research to provide more insights into understanding of multiple aspects of MSC-EVs in HCEC protection. An important facet to be defined is the mechanisms of the regenerative potential of MSC-EVs on HCECs. As previously described, many studies have shown that MSCs can protect tissues from damage through the paracrine actions of EVs. The content of an EV is dependent on its origin, size, and the route of biogenesis. EVs are rich with protein such as platelet-derived growth factor (PDGF), organelles, lipids, mRNAs, microRNAs, mitochondria, and cytokines [86]. The presence of a complex cargo within EVs results in a multilevel modulation of cell functions in the recipient cells [87].

For example, PDGF is a putative antiapoptotic factor that seems to be able to stimulate HCEC growth [88]. PDGF stimulates the proliferation of megakaryocytes, erythrocytes, leukocytes, and their progenitors, presumably through the multiple endogenous growth factors released from mesenchymal stem/stromal cells [89]. Joyce et al. have provided evidence that HCEC in vivo is arrested in G1-phase of the cell cycle [90], but they have also demonstrated that HCECs can proliferate in vitro in response to growth-promoting agents [88]. Their study compared the effect of several growth-promoting agents on proliferation of HCECs from young and older donors. Nerve growth factor did not enhance proliferation above basal levels, regardless of donor age. Epidermal growth factor (EGF) moderately stimulated proliferation in cells from younger donors but not in HCECs from older donors. On the other side, PDGF and bovine pituitary extract stimulated proliferation above the level induced by EGF, while the combination of these two agents had a strong additive effect, notably increasing cell proliferation above that achieved with either factor alone. Of the growth-promoting agents tested, fetal bovine serum was the one that stimulated the greatest proliferation of HCECs, in both younger and older donors. Proliferation in the presence of multiple mitogens ceased when confluence was reached, indicating the formation of a contact-inhibited monolayer.

Another component that may be involved in proliferative potential of EVs is encapsulated mitochondria. MSCs are shown to transfer mitochondria to the recipient cells in different ways: encapsulated within EVs, via cell-to-cell direct communication through tunnelling nanotubes, or through direct release of “naked” mitochondria into the extracellular microenvironment [86]. The organelle incorporates into the endogenous mitochondrial network of the damaged cell that needs to be rescued, reestablishing its bioenergetic homeostasis [91]. The incorporation of mitochondria within released microvesicles has been widely studied in MSCs, where once internalized, mitochondria containing microvesicles can rescue cells from damage or act as reprogramming agents [90]. Recent findings suggest that mitochondrial transfer may have a key role in protection of ocular cells, including corneal epithelial cells and RGCs. Jiang et al. demonstrated that MSCs can efficiently donate functional mitochondria and protect corneal epithelial cells from oxidative stress-induced damage through tunnelling nanotube (TNT) formation. Furthermore, oxidative inflammation improved the efficiency of mitochondrial transfer from MSCs to stressed corneal epithelial cells and increased TNT formation that is regulated by the NF-*κ*B signaling pathway [92]. Jiang et al. more recently provided evidences that intravitreal transplanted induced pluripotent stem cells-derived MSCs (iPSC-MSCs) can effectively donate functional mitochondria to RGCs and protect against mitochondrial damage-induced RGC loss [93]. Mitochondrial transfer from MSCs could provide a novel mechanism of protection also for the corneal endothelium.

Additionally, it seems that a proinflammatory environment can enhance MSC-EV production. As discussed, MSCs displayed a high potential due to secretion of therapeutic factors, both free and conveyed within EVs, and collectively termed secretome. Ragni et al. tried to characterize adipose-derived MSC- (ASC-) secreted factors and EV-miRNAs and their modulation after IFN*γ* preconditioning in joint disease [94]. Given the assortment of soluble factors and EV-miRNAs, ASC secretome showed the ability to promote cell motility and modulate inflammatory and degenerative processes. Preconditioning is able to increase this ability, suggesting inflammatory priming as an effective strategy to obtain a more potent clinical product which use should always be driven by the molecular mark of the target pathology.

In summary, theoretical framework and results of our study in the corneal endothelium setting are promising, but further research will be needed in order to better understand the key components of MSC-EVs that are acting on the protection and damage restoration of HCECs.

For future clinical application of our results, it should be considered that conventional manufacturing approaches of BM-MSCs are challenged by their limited capacity to proliferate and their loss of differentiation potential. Indeed, human iPSC-MSCs may represent a better option to overcome these limitations and be more indicated for tissue regeneration [95]. iPSC-MSCs may therefore represent an unlimited cell source for the production of EVs in large scale [96].

## 5. Conclusions

In conclusion, in our study, we showed that *in vitro* MSC-EV administration on HCECs has been able to induce proliferation and migration of damaged HCECs and was effective in inhibiting cell apoptosis. This work could represent a valid starting point to explore the effect of MSC-EVs on this type of model in order to make it possible in the future to exploit MSC-EV treatment *in vivo* in patients with corneal endothelial dysfunctions. Our finding suggests that human MSC-EVs may represent a novel therapeutic approach that could lead to an increasing independence of eye banks that could be of great importance to reduce the number of worldwide corneal blindness. Anyway, more studies are useful to determine their possible therapeutic value and the mechanisms involved, so they should be actively pursued in the future.

## Figures and Tables

**Table 1:** Clinical and biological information of patients undergoing penetrating keratoplasty. In the table are listed the clinical and biological aspects of patients from which we received corneal buttons.

Sex	Age	Diagnosis	Surgical procedure	Medical history	Ophthalmological therapy
F	75	Ocular hypertonia of traumatic etiology	Enucleation	Allergies (indomethacin, tramadol, ciprofloxacin), arterial hypertension	Topical therapy: timolol, diclofenac
F	35	Keratoconus	PKP		Topical therapy: hydrocortisone
F	66	Fuchs' endothelial corneal dystrophy (FECD)	PKP	Smoking, diabetes mellitus type II, arterial hypertension	Topical therapy: loteprednol (od)
F	47	Corneal leukoma	PKP	Postsurgical hypothyroidism	
M	75	Corneal leukoma	PKP	Arterial hypertension, benign prostatic hypertrophy	Systemic therapy: acetazolamide topical therapy: brinzolamide, timolol
M	78	Corneal leukoma	PKP	Left eye trauma at 20 years old, arterial hypertension, diabetes mellitus type II, prostatic cancer	
F	83	FECD	PKP	Allergies (penicillin, metamizole), arterial hypertension, hypercholesterolemia, hypothyroidism, depression	Topical therapy: indomethacina, bromfenac, edenorm
F	47	FECD	PKP	Postsurgical hypothyroidism	
M	81	FECD	PKP	Arterial hypertension COPD, diabetes mellitus type II, benign prostatic hypertrophy	Topical therapy: netilmicin, dexamethasone, ofloxacin
M	40	Keratoconus	PKP	Allergies (pollen)	
F	40	Pellucid marginal degeneration	PKP	Allergies (pollen)	
F	77	FECD	PKP	Diabetes mellitus type II, diabetical neuropathy, acute myocardial infarction, kidney failure treated with transplant, HCV+	Topical therapy: brinzolamide, timolol, brimonidine
M	80	FECD	PKP	Arterial hypertension, keratoconus in the both eyes	Topical therapy: cloramphenicol dexamethasone, bluyal a
F	70	Corneal leukoma	PKP	Pontomesencephalic cavernoma	
F	72	Corneal leukoma	PKP	Arterial hypertension, asthma, ulcerative rectocolitis	Topical therapy: trehalose, clobetasone
F	31	Keratoconus	PKP	Down's syndrome, seasonal affective disorder	
M	71	FECD	PKP		
M	67	FECD	PKP	Parkinson's disease, gastroesophageal reflux disease, benign prostatic hypertrophy, biliary stones, paranoid psychosis	
F	24	Corneal leukoma	PKP	Acute myeloid leukemia in remission, seasonal affective disorder	
M	75	Late failure of transplanted cornea	PKP	Arterial hypertension, benign prostatic hypertrophy	
M	78	Late failure of transplanted cornea	PKP	Diabetes mellitus type II	
F	21	Corneal leukoma (chemical burn)	PKP	Seasonal affective disorder	
F	66	Corneal leukoma	PKP	Facio-scapular-humeral muscular dystrophy	

COPD: chronic obstructive pulmonary disease; FECD: Fuchs' endothelial corneal dystrophy; HCV: hepatitis C virus; PKP: penetrating keratoplasty.

## Data Availability

The data used to support the findings of this study are available from the corresponding author upon request.

## Abbreviations

AMD: Age-related macular degeneration
EGF: Epidermal growth factor
EVs: Extracellular vesicles
FECD: Fuchs' endothelial corneal dystrophy
HCECs: Human corneal endothelial cells
iPSC-MSCs: Induced pluripotent stem cells-derived MSCs
MSCs: Mesenchymal stem cells
MSC-EVs: Extracellular vesicles derived from mesenchymal stem cells
PDGF: Platelet-derived growth factor
PKT: Penetrating keratoplasty
PLK: Posterior lamellar keratoplasty
RGCs: Retinal ganglion cells
TM: Trabecular meshwork
TNTs: Tunnelling nanotubes.
